# Cricopharyngeal myotomy for cricopharyngeus muscle dysfunction after esophagectomy

**DOI:** 10.1186/s40792-020-00961-3

**Published:** 2020-08-12

**Authors:** Shinichiro Kobayashi, Ken Taniguchi, Fujinobu Tanaka, Shigeto Maeda, Takanori Hirayama, Takayuki Tokunaga, Hiroaki Takeshita, Shiro Miura, Tamotsu Kuroki

**Affiliations:** 1 Department of Surgery, National Hospital Organization Nagasaki Medical Center, Kubara 2-1001-1, Ohmura, Nagasaki, 8568562 Japan; 2 Department of Surgery, Nagasaki Harbor Medical Center, Nagasaki, Japan; 3 Department of Head and Neck Surgery, National Hospital Organization Nagasaki Medical Center, Nagasaki, Japan; 4 Department of Pathology, National Hospital Organization Nagasaki Medical Center, Nagasaki, Japan

**Keywords:** Esophagectomy, Cricopharyngeus muscle dysfunction, Cricopharyngeus myotomy

## Abstract

**Background:**

Pharyngoesophageal dysphagia sometimes develops after esophagectomy. However, severe dysphagia after esophagectomy due to cricopharyngeus muscle dysfunction is a rare complication. There are no recommended clinical treatments for cricopharyngeus muscle dysfunction after esophagectomy. We report a case of myotomy for cricopharyngeus muscle dysfunction after esophagectomy.

**Case presentation:**

A 75-year-old man with mild dysphagia diagnosed with advanced esophageal cancer by esophagogastroduodenoscopy at a clinic was admitted to our hospital. He had occasional mild dysphagia when he swallowed solid foods. After chemotherapy, the patient underwent minimally invasive esophagectomy with regional lymph node dissection and was reconstructed with a gastric conduit and cervical anastomosis by the retrosternal route. Aspiration pneumonia developed after esophagectomy without paralysis of the vocal cords. In esophagoscopy, there was no stricture around the anastomosis. However, severe pharyngoesophageal dysphagia with cricopharyngeus muscle dysfunction was revealed by videofluoroscopic examination. Bilateral cricopharyngeal myotomy was performed because balloon dilations had failed. The histological findings revealed atrophy and fibrosis of the cricopharyngeus muscle fibers. Pharyngoesophageal dysphagia improved immediately after myotomy. The patient swallowed solid food easily without dysphagia 12 months after myotomy.

**Conclusion:**

Dysphagia after esophagectomy was worsened by cricopharyngeus muscle dysfunction. Cricopharyngeus myotomy may lead to long improvement of pharyngo-oesophageal dysphagia after esophagetomy.

## Background

Dysphagia is a common predominant symptom after esophagectomy [1–4]. Cervical anastomosis and paralysis of recurrent laryngeal nerve sometimes cause pharyngeal swallowing dysfunction and aspiration pneumonia [5, 6]. Cricopharyngeus muscle dysfunction is a disorder caused by failure of the upper esophageal sphincter (UES) to relax during swallowing, thereby causing oropharyngeal dysphagia [7]. However, severe dysphagia due to cricopharyngeus muscle atrophy after esophagectomy is a rare complication. Thus, there are no recommended clinical treatments for severe dysphagia because of cricopharyngeus muscle hypertrophy after esophagectomy.

We report a successful case of cricopharyngeal myotomy for cricopharyngeus muscle dysfunction after esophagectomy.

## Case presentation

**Fig. 1:**
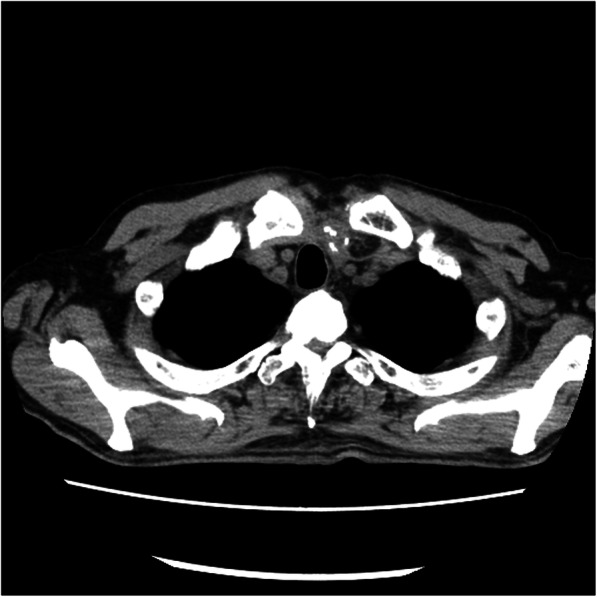
Computed tomography examination. No abnormalities around the cervical anastomosis after esophagectomy were observed

**Fig. 2:**
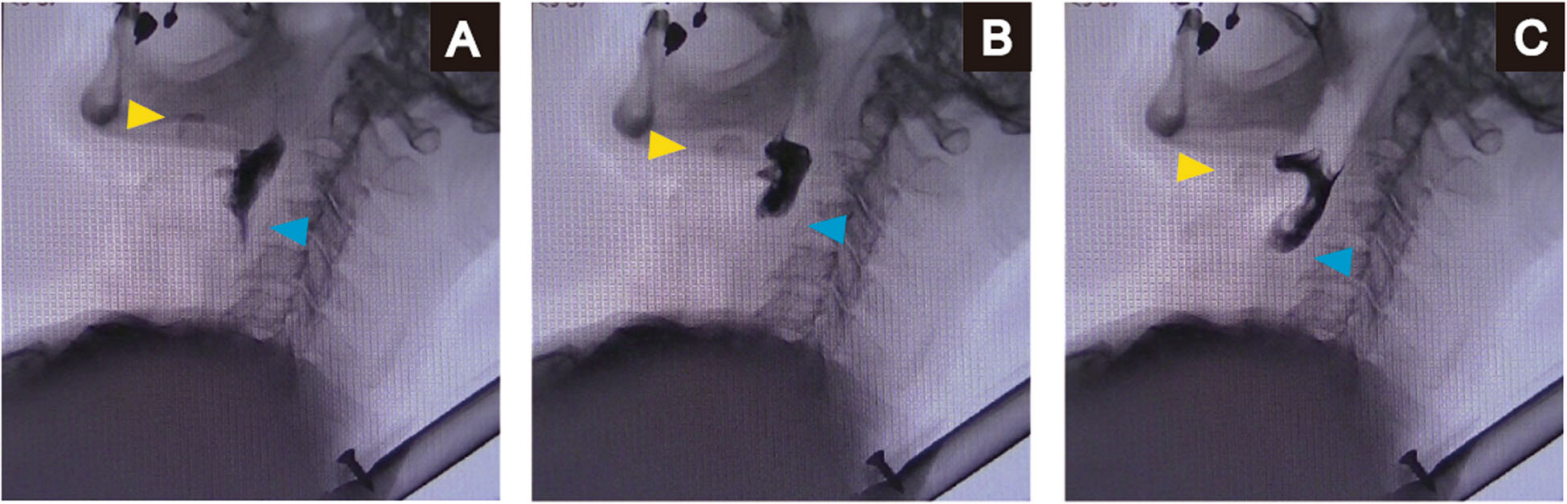
Swallow functional test before cricopharyngeal myotomy. **a** Laryngeal penetration occurred during laryngeal elevation in the pharyngeal phase. **b** The bolus did not pass entirely through the upper esophageal sphincter in the esophageal phase. **c** Breathing was reinitiated with a massive amount of pharyngeal residue. Yellow arrow; Hyoid bone, Blue arrow; Esophageal orifice

**Fig. 3:**
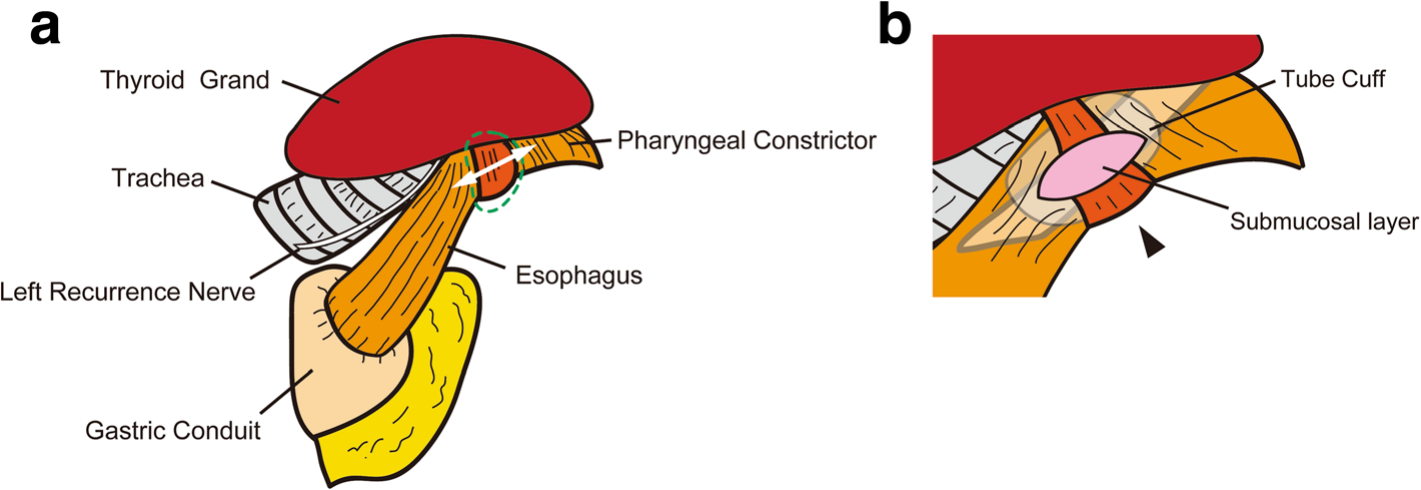
Schematic of cricopharyngeal myotomy. **a** Fibrosis occurred in the cricopharyngeus muscle (green dots). The resection line (white line) was demonstrated lateral cricopharyngeus muscle. **b** When the bilateral cricopharyngeus muscles (arrow) were resected, a disposable endotracheal tube (Inner diameter: 5.5 mm) was inserted in pharingoesophageal region, and the cuff was gradually dilated. The myotomy allowed the dilated mucosa

**Fig. 4:**
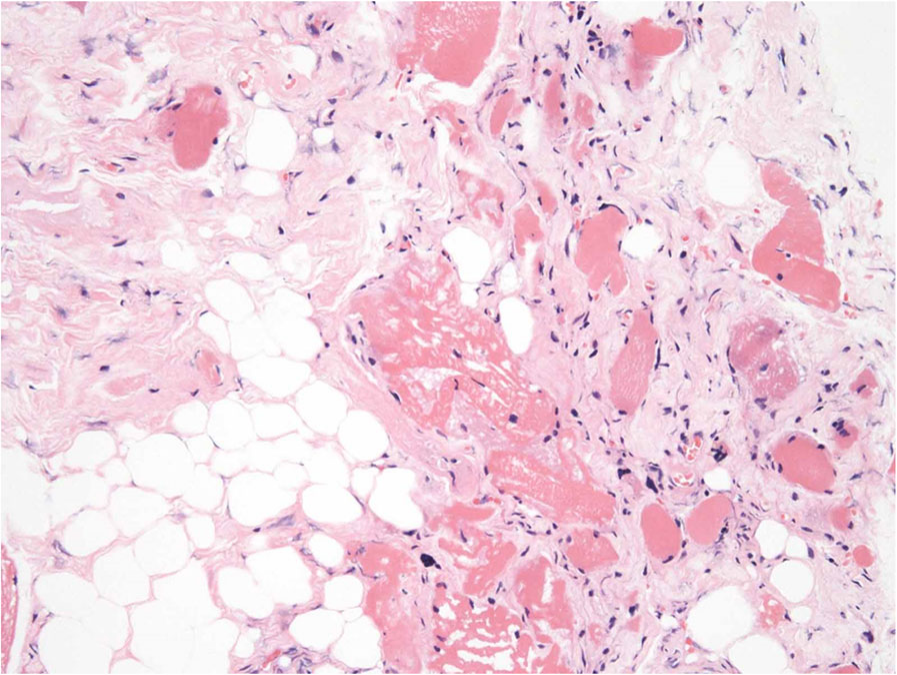
Histopathological findings. Diffuse atrophy and fibrosis of the cricopharyngeus muscle fibers developed

**Fig. 5:**
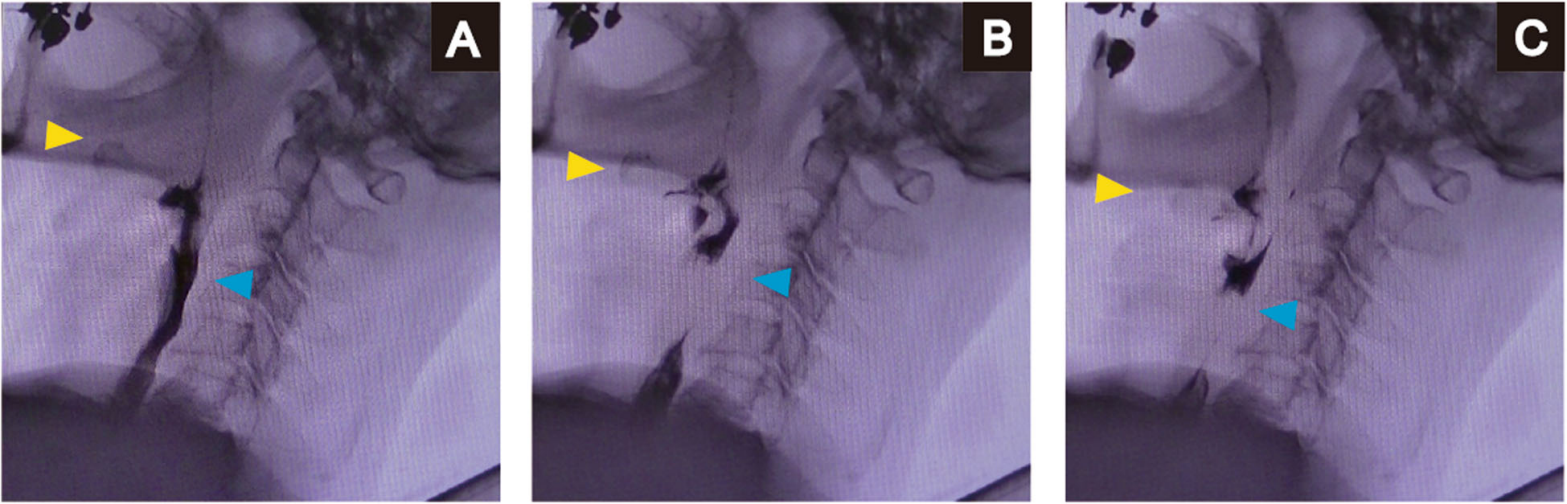
Swallow functional test after cricopharyngeal myotomy. **a** The bolus easily entered the esophagus during laryngeal elevation in the pharyngeal phase. **b** Slight laryngeal penetration occurred in the esophageal phase. **c** Breathing was reinitiated with a small amount of pharyngeal residue. Yellow arrow; Hyoid bone, Blue arrow; Esophageal orifice

A 75-year-old man was admitted to a clinic with mild dysphagia. His past and family histories were unremarkable. Esophagogastroduodenoscopy (EGD) at the clinic revealed a tumor of the thoracic esophagus. He was referred to our hospital for further examination. His physical findings were normal. All laboratory data were within normal limits. Tumor marker levels were all within normal limits. An EGD revealed a type II tumor, 20 mm in diameter, of the thoracic esophagus. The histological findings of biopsy specimens from the tumor showed squamous cell carcinoma. Computed tomography (CT) showed swollen lymph nodes and mediastinum metastasis without invasion to the adjacent organs. The esophageal carcinoma was clinically diagnosed as T2N1M0 (StageII) according to the eighth edition of the Union for International Cancer Control [8]. The patient underwent preoperative chemotherapy, followed by minimally invasive esophagectomy with regional lymph node dissection and gastric conduit reconstruction by the retrosternal route following the Esophageal cancer practice guidelines 2017 edited by the Japan Esophageal Society [9]. The esophageal carcinoma was finally diagnosed as ypT1bN0M0 (ypStageI) [10]. The patient did not developed hoarseness. However, pharyngoesophageal dysphagia developed, and he was unable to swallow anything immediately after the esophagectopmy. In laryngoscopy, there was no paralysis of the vocal cords, although left vocal code weakened on postoperative day (POD) 7. In EGD, there was no stricture around the anastomosis. In CT scan, there was also no compartmental tissue around the anastomosis (Fig. 1). Severe supraglottic penetration with cricopharyngeus muscle dysfunction was revealed in videofluoroscopic examination (Fig. 2). Although balloon dilations around the pharyngoesophageal junction had been performed several times, cricopharyngeus muscle dysfunction remained, and the patient developed aspiration pneumonia. Bilateral cricopharyngeal myotomy was performed for cricopharyngeus muscle dysfunction on POD 43. Access to pharingoesophageal region was obtained through an oblique cervical incision bordering the anterior edge of the sternocleidomastoid muscle. The region was exposed by retracting the carotid sheathes laterally and the trachea and larynx medially. The incision lines were placed in bilateral cricopharyngeus muscles without the injury of the recurrent nerves. The incisions to the cricopharyngeus muscle were carried down to the mucosa and were extended caudally by 30 mm on to the esophagus (Fig. 3a). A disposable plastic endotracheal tube (Inner diameter: 5.5 mm) was inserted in pharingoesophageal region, and the cuff was gradually dilated in the myotomy. The myotomy allowed the dilated mucosa to protrude freely through the incision (Fig. 3b). The histological findings revealed atrophy and fibrosis of the cricopharyngeus muscle fibers (Fig. 4). Pharyngoesophageal dysphagia improved immediately after myotomy. Supraglottic penetration with cricopharyngeus muscle dysfunction was improved in videofluoroscopic examination (Fig. 5). The patient swallowed solid food easily without dysphagia 12 months after myotomy.

## Discussion

Cricopharyngeus muscle atrophy and spasm cause cricopharyngeus muscle dysfunction. Atrophy of the cricopharyngeal muscle especially prolongs deglutition and causes severe aspiration [11]. Cricopharyngeus muscle atrophy is associated with paralysis or various systemic diseases, including motor neuron disease, general granulomatous disease, and muscle atrophy [11, 12]. Structural and functional pharyngeal changes occur with age. Cricopharyngeal abnormalities sometimes develop in adults without dysphagia over 70 years old [13]. In the present case, mild dysphagia before esophagectomy, the chief complaint, was likely induced by latent cricopharyngeus muscle dysfunction with cricopharyngeus muscle atrophy but not stenosis of esophageal cancer.

In esophagectomy, the most general gastric reconstruction is gastric conduit [9]. Cervical anastomosis and paralysis of the recurrent laryngeal nerve sometimes cause pharyngeal swallowing dysfunction and aspiration pneumonia [3, 4]. Vocal fold immobility due to injury to the recurrent nerve during esophagectomy causes penetrated materials to be easily aspirated to the trachea [6]. Moreover, several biomechanical abnormalities in swallowing can develop after esophagectomy [6, 14]. These abnormalities, which include delayed onset of swallowing, reduced hyoid or hyolaryngeal elevation, and reduced UES opening, are demonstrated by videofluoroscopic examination and high-resolution manometry [7, 15]. The reconstruction route may also cause swallowing abnormalities [1]. The hyoid or hyolaryngeal elevation is reduced in retrosternal reconstruction due to adhesion to the front of the trachea [1]. The UES opening is also reduced in retrosternal reconstruction [1]. In the present case, the cricopharyngeus muscle was not directly accessed in esophagectomy. In the dissection of cervical paraesophageal lymph nodes, energy devices were not used. Retrosternal reconstruction after esophagectomy may have caused severe pharyngoesophageal dysphagia in addition to cricopharyngeus muscle atrophy before esophagectomy.

In the field of laryngology, cricopharyngeal myotomy has been successfully employed to treat cricopharyngeus muscle dysfunction caused by neurological disease or surgery for head and neck cancer [16, 17]. Fujita et al. reported that cricopharyngeal myotomy is effective as an operative rehabilitation procedure for severe dysphagia after esophagectomy [18]. Endoscopic cricopharyngeal myotomy has recently been reported as a new approach that appears to be as effective but less morbid than classical open surgery [16]. Botulinum toxin A injection is a less invasive therapy for cricopharyngeus dysfunction, although dysphagia reoccurs more often with this therapy than with cricopharyngeus dilation and myotomy [19]. Although dilation is likely to provide temporary benefit, surgical myotomy provides long remission [20]. In the present case, cricopharyngeal myotomy provided longer relief of cricopharyngeus muscle dysfunction, although cricopharyngeus dilation also failed to improve cricopharyngeus muscle dysfunction.

## Conclusions

In conclusion, we have described a case of cricopharyngeus muscle dysfunction after esophagectomy. Swallowing functional tests should be used to examine patients with dysphagia before esophagectomy. Cricopharyngeal myotomy may improve pharyngoesophageal dysphagia after esophagectomy.

## Data Availability

Not applicable.

## Abbreviations

UES: upper esophageal sphincter
EGD: Esophagogastroduodenoscopy
CT: Computed tomography
POD: postoperative day

## References

[1] Kato H, Miyazaki T, Sakai M, Sano A, Tanaka N, Kimura H (2007). Videofluoroscopic evaluation in oropharyngeal swallowing after radical esophagectomy with lymphadenectomy for esophageal cancer. Anticancer Res.

[2] Kumai Y, Samejima Y, Watanabe M, Yumoto E (2017). Videofluoroscopic evaluation of pharyngeal swallowing dysfunction after esophagectomy with three-field lymph node dissection. Eur Arch Otorhinolaryngol.

[3] Kumai Y, Miyamoto T, Matsubara K, Samejima Y, Yoshida N, Baba H (2019). Determining the efficacy of the Chin-down maneuver following Esophagectomy with Fiberoptic endoscopic evaluation of swallowing. Arch Phys Med Rehabil.

[4] Yuen MTY, Tsang RK, Wong IYH, Chan DKK, Chan FSY, Law SYK. Long-term pharyngeal dysphagia after esophagectomy for esophageal cancer-an investigation using videofluoroscopic swallow studies. Dis Esophagus. 2019;32(1). 10.1093/dote/doy068. 10.1093/dote/doy068 30085000

[5] Lee SY, Cheon HJ, Kim SJ, Shim YM, Zo JI, Hwang JH (2016). Clinical predictors of aspiration after esophagectomy in esophageal cancer patients. Support Care Cancer.

[6] Kaneoka A, Yang S, Inokuchi H, Ueha R, Yamashita H, Nito T (2018). Presentation of oropharyngeal dysphagia and rehabilitative intervention following esophagectomy: a systematic review. Dis Esophagus.

[7] Okumura T, Shimada Y, Watanabe T, Nakamichi N, Nagata T, Tsukada K (2016). Functional outcome assessment of swallowing (FOAMS) scoring and videofluoroscopic evaluation of perioperative swallowing rehabilitation in radical esophagectomy. Surg Today.

[8] Rice TW, Ishwaran H, Blackstone EH, Hofstetter WL, Kelsen DP, Apperson-Hansen C (2016). Recommendations for clinical staging (cTNM) of cancer of the esophagus and esophagogastric junction for the 8th edition AJCC/UICC staging manuals. Dis Esophagus.

[9] Kitagawa Y, Uno T, Oyama T, Kato K, Kato H, Kawakubo H (2019). Esophageal cancer practice guidelines 2017 edited by the Japan esophageal society: part 2. Esophagus..

[10] Rice TW, Ishwaran H, Kelsen DP, Hofstetter WL, Apperson-Hansen C, Blackstone EH (2016). Recommendations for neoadjuvant pathologic staging (ypTNM) of cancer of the esophagus and esophagogastric junction for the 8th edition AJCC/UICC staging manuals. Dis Esophagus.

[11] Bakheit AM (2001). Management of neurogenic dysphagia. Postgrad Med J.

[12] Wang J, Li W, Li Y, Jin X, Niu Y, Tian X (2018). A novel endoscopic surgery for dysphagia after stroke. Surg Endosc.

[13] Yin T, Jardine M, Miles A, Allen J (2018). What is a normal pharynx? A videofluoroscopic study of anatomy in older adults. Eur Arch Otorhinolaryngol.

[14] Martin RE, Letsos P, Taves DH, Inculet RI, Johnston H, Preiksaitis HG (2001). Oropharyngeal dysphagia in esophageal cancer before and after transhiatal esophagectomy. Dysphagia..

[15] Matsubara K, Kumai Y, Miyamoto T, Samejima Y, Yoshida N, Baba H, et al. The effect of a chin-down maneuver after esophagectomy on oropharyngeal swallowing pressure measured using high-resolution manometry. Auris Nasus Larynx. 2019. 10.1016/j.anl.2019.06.001 31204048

[16] Ashman A, Dale OT, Baldwin DL (2016). Management of isolated cricopharyngeal dysfunction: systematic review. J Laryngol Otol.

[17] Sung A, Lee KW (2020). Cricopharyngeal myotomy for delayed Cricopharyngeal dysfunction after head and neck surgery - case report. BMC Surg.

[18] Fujita H, Hirano M, Kakegawa T, Yamana H, Tanaka S (1991). Surgical treatment for aspiration following esophageal reconstruction--a report of two cases and the techniques involved. Jpn J Surg.

[19] Kocdor P, Siegel ER, Tulunay-Ugur OE (2016). Cricopharyngeal dysfunction: a systematic review comparing outcomes of dilatation, botulinum toxin injection, and myotomy. Laryngoscope..

[20] Marston AP, Maldonado FJ, Ravi K, Kasperbauer JL, Ekbom DC (2016). Treatment of oropharyngeal dysphagia secondary to idiopathic cricopharyngeal bar: surgical cricopharyngeal muscle myotomy versus dilation. Am J Otolaryngol.

